# Preparation and adsorption properties of magnetic chitosan/sludge biochar composites for removal of Cu^2+^ ions

**DOI:** 10.1038/s41598-023-46815-4

**Published:** 2023-11-28

**Authors:** Meng Zhang, Yunqing Liu, Zhizhen Yin, Dan Feng, Hui Lv

**Affiliations:** Key Laboratory of Pollutant Chemistry and Environmental Treatment, School of Resources and Environment, Yili Normal University, Xinjiang, 835000 Yining China

**Keywords:** Environmental sciences, Environmental impact

## Abstract

The magnetic chitosan/sludge biochar composite adsorbent was prepared using chitosan, Fe_3_O_4_, and sludge biochar as raw materials. The composite adsorbent was able to achieve rapid solid–liquid separation under an applied magnetic field. The morphology and microstructure of the composite adsorbent were characterized by FTIR, XRD, SEM, VSM, and BET analysis. The adsorption performance of the composite adsorbent on Cu^2+^ was investigated through static adsorption experiments, and the effects of adsorbent dosage, initial concentration of Cu^2+^, initial pH of the solution, and adsorption temperature on the adsorption efficiency of Cu^2+^ were discussed. The results showed that chitosan and Fe_3_O_4_ were successfully loaded on sludge biochar. When the initial concentration of Cu^2+^ was 30 mg/L, the dosage of the magnetic chitosan/sludge biochar composite material was 0.05 g, the adsorption time was 180 min, pH was 5, and the temperature was room temperature, the maximum removal rate of Cu^2+^ reached 99.77%, and the maximum adsorption capacity was 55.16 mg/g. The adsorption kinetics and adsorption isotherm data fitted well with the pseudo-second-order kinetic model and Langmuir adsorption isotherm model, indicating that the adsorption process was chemisorption with monolayer coverage.

## Introduction

With the rapid growth of the Chinese economy and the flourishing development of various industries, numerous types of pollution have emerged. Among these, the issue of heavy metal pollution in water bodies has always been a focal point of concern as well as a challenging problem in water pollution control. Wastewater containing heavy metals generated during industrial production processes, such as mining, mechanical manufacturing, and the chemical industry, is one of the most significant sources of pollution in water bodies. These pollutants pose a severe threat to human life and health. Heavy metals, including Cr^6+^, Pb^2+^, Hg^2+^, V^2+^, and Cu^2+^, are particularly harmful contaminants found in water bodies. Among them, water pollution caused by Cu^2+^ primarily originates from industries such as electroplating, non-ferrous metal processing, and copper mining. When the copper concentration in water reaches 0.01 mg/L, it significantly hinders the self-purification process of water bodies. At levels exceeding 3.0 mg/L, it generates an unpleasant odor, and when the concentration exceeds 15 mg/L, the water becomes unfit for consumption^1^. The United States Environmental Protection Agency (EPA) has set the allowable limit of Cu^2+^ in industrial wastewater at 1.3 mg per liter. Additionally, the World Health Organization (WHO) has established the allowable limit of Cu^2+^ in drinking water at 1.5 mg/L^2^. Consequently, it is crucial to develop more efficient and environmentally friendly adsorbent materials to tackle Cu^2+^ pollution in water bodies.

There are many methods for removing heavy metals from polluted water bodies, including chemical precipitation, electrochemical methods, membrane separation, ion exchange, biological methods, and adsorption, etc^3^. Among them, adsorption has the advantages of simple operation, good effect, clean process, easy regeneration, and no secondary pollution, making it one of the most promising technologies for heavy metal wastewater treatment. However, commonly used adsorbents such as activated carbon, zeolite, and natural clay have low adsorption capacity and poor adsorption selectivity, which often result in unsatisfactory results in the treatment of heavy metal wastewater.

Currently, the total amount of sludge produced by urban sewage treatment plants in China has been rapidly increasing at a growth rate exceeding 10% since 2007. The sludge contains a high content of organic matter (30% ~ 70%) and is inexpensive and readily available. Utilizing sludge to produce biochar is considered a promising disposal method^4^. Sludge biochar is a solid material generated from the anaerobic or oxygen-limited high-temperature pyrolysis of sludge. Due to its abundant pore structure, aromatic layer structure, and low cost, biochar is regarded as a novel adsorbent with great potential in the field of water treatment.

Chitosan is a natural alkaline polysaccharide and is the product of deacetylation of chitin^5^. Chitosan molecules contain many hydroxyl and amino groups, which can form hydrogen bonds, electrostatic interactions, and van der Waals forces with heavy metal ions, thereby achieving the purpose of adsorbing heavy metal ions. Chitosan is widely used in the removal of heavy metal ions due to its advantages such as biodegradability, antibacterial properties, hydrophilicity, renewability, and environmental friendliness. To enhance adsorption performance, magnetic adsorbents such as Fe_3_O_4_ magnetic particles and nanoparticles are often loaded on the surface of chitosan and applied in the treatment of heavy metal wastewater^6^.

Chao et al.^7^ prepared chitosan-modified magnetic nanoparticles using a reverse suspension cross-linking method to address the issue of molybdenum exceeding the standard in mining wastewater. This material was used to adsorb molybdenum (VI) from water, and the results showed that the theoretical maximum adsorption capacity was determined to be 35.54 mg/g. Even after three cycles of regeneration, the adsorption rate of chitosan-modified magnetic nanoparticles for Mo (VI) remained above 90%. The chitosan-modified magnetic nanoparticles are a fast and efficient method for removing Mo (VI). They have good reusability, strong magnetic properties, and are suitable for solid–liquid magnetic separation.

Zheng et al.^8^ synthesized recyclable magnetic biochar functionalized with chitosan and ethylenediaminetetraacetic acid (E-CMBC) to investigate its adsorption performance for Pb (II) in aqueous solutions and the underlying potential adsorption mechanism. The results showed that under pH 3.0 conditions, the removal rate of Pb (II) was significantly increased to 156.68 mg/g compared to the unmodified original biochar (10.90 mg/g). The magnetic intensity of E-CMBC was measured to be 3.1 emu/g, suggesting that the consumed E-CMBC could be separated from water using an external magnet. Regeneration studies showed that after three cycles of adsorption–desorption, the recovery rate of the adsorbent was 78.60%, and the adsorption capacity retention rate was 97.26%. In conclusion, E-CMBC is a novel, recyclable, and efficient adsorbent for Pb (II) removal.

Li et al.^9^ prepared amino-thiourea modified chitosan-magnetic biochar composite (TMBC) for the efficient removal of Cd (II) from wastewater. The synthesized material was characterized and its adsorption mechanism and thermodynamics were extensively studied. Adsorption experiments revealed that TMBC exhibited higher affinity for Cd (II) compared to magnetic biochar composite, biochar, and other carbon-based adsorbents. The adsorption process of Cd (II) followed a pseudo-second-order kinetic model. The maximum adsorption capacities based on the Langmuir model at 298, 308, and 318 K were 93.72, 121.9, and 137.3 mg/g, respectively. Kang et al.^10^ prepared sewage sludge-based biochar loaded with nanoscale zero-valent iron (NZVI) under 700 °C (nBC700) conditions for the removal of Cr (VI) and Cu (II). The results showed that the addition of NZVI greatly enhanced the adsorption capacity of biochar for Cu (II) and Cr (VI), with increases of 251.96% and 205.18%, respectively.

In summary, it is particularly important to find an adsorbent with high adsorption capacity, economical, and environmentally friendly for treating wastewater contaminated with heavy metals. Using dehydrated sludge as raw material, converting it into sludge-based biochar through high-temperature pyrolysis, preparing highly efficient composite materials by loading a small amount of CTS and Fe_3_O_4_, and selectively using them for treating heavy metal wastewater not only improves the added value of the sludge-based biochar, but also makes the prepared composite adsorbent have a certain degree of magnetism. The sludge was separated from water by applying an external magnetic field to reduce the secondary pollution of water bodies. Meanwhile, the chitosan on the surface of the composite material has active groups such as amino and hydroxyl groups, and the nano-magnetic powder contained in the composite material has a large specific surface area and is uniformly attached to the surface of the sludge biochar, which improves the adsorption performance. By studying the optimal conditions for the adsorption of heavy metal ions by composite materials, it provides an important theoretical foundation and scientific basis for more efficient removal of heavy metal ions from wastewater.

In this paper, a composite material was prepared using chitosan (CTS), iron (III) oxide (Fe_3_O_4_), and sewage sludge-based biochar (SBC). FTIR, XRD, SEM, VSM, and BET techniques were employed to characterize their functional groups, structures, morphologies, magnetic properties, specific surface areas, and pore volumes. The effect of magnetic chitosan/sludge-based biochar (Fe_3_O_4_@CTS/SBC) composites on the removal of Cu^2+^ from simulated wastewater was investigated by controlling variables such as initial concentration of Cu^2+^, adsorbent dosage, pH, temperature and other adsorption properties. The adsorption characteristics of Fe_3_O_4_@CTS/SBC on Cu^2+^ were investigated by adsorption isotherms, adsorption kinetics and adsorption thermodynamics.

## Material and methods

### Reagents and materials

All chemical reagents were of analytical grade, and the solutions used in this study were prepared with deionized water. Chitosan was purchased from Shanghai Aladdin Biochemical Technology Co., Ltd; Iron oxide was purchased from Beijing Nan Shang Le Chemical Factory; Glutaraldehyde 50%, sodium hydroxide and hydrochloric acid were purchased from Tianjin Fuchen Chemical Reagent Factory; Acetic acid and ammonia solution were purchased from Tianjin Beilian Fine Chemical Development Co., Ltd; 1,3-dicyclohexyl-2-methylidenecarboxamide was purchased from Xi'an Chemical Reagent Factory; Anhydrous ethanol and anhydrous copper sulfate were purchased from Tianjin Damao Chemical Reagent Factory; Triethylenetetramine citrate was purchased from Shanghai Sihewi Chemical Co., Ltd.

### Preparation method of adsorbent

#### Preparation of Sewage Sludge Biochar (SBC)

Sludge biochar (SBC) was prepared from the remaining sludge of a sewage treatment plant in Yining City. The sludge was first placed under ventilated conditions to dry naturally drying. It was then thoroughly ground and screened through an 80-mesh sieve size for later use. Next, an appropriate amount of dried sludge was removed from the pretreated sludge and placed in a crucible. The crucible was sealed with tinfoil and placed in a muffle furnace for pyrolysis by thermal cracking. The pyrolysis process consisted of seven steps: 20 °C to 200 °C, heating time 1 h, holding time 1 h at 200 °C; 200 °C to 300 °C, heating time 1 h, holding time 1 h at 300 °C; 300 °C to 400 °C heating time 1 h, holding time 1 h at 400 °C; 400 °C to 500 °C, heating time 1 h, holding time 1 h at 500 °C; 500 °C to 300 °C, cooling time half an hour; 300 °C to 200 °C, cooling time half an hour; and 200 °C to 25 °C, cooling time half an hour. This process produced the desired sewage sludge biochar. In order to remove the excess inorganic substances and ash content, the sewage sludge biochar was treated by acid washing with dilute hydrochloric acid (biochar: dilute hydrochloric acid) in the ratio of 1:10. After stirring on a magnetic stirrer for 1 h, the mixture was filtered and washed with water until neutral. Finally, the biochar was dried and reserved for further use.

#### Preparation of magnetic chitosan (Fe_3_O_4_ @CTS/SBC)

0.5 g of chitosan powder was weighed using an analytical balance and added to 20 mL of acetic acid at 2% volume fraction. The mixture was stirred thoroughly at room temperature with a magnetic stirrer until chitosan was completely dissolved to obtain chitosan acetic acid solution. Added 0.2 g Fe_3_O_4_ and stirred well, ultrasonicated for 30 min and dispersed evenly. Added 3 mL of 25% glutaraldehyde and placed the reaction mixture in a 50 °C water bath for cross-linking reaction for 1 h. After completion of the cross-linking reaction, the pH was adjusted to 8 by gradually dropping NaOH solution. Further cross-linking reaction was carried out by continuing heating in a water bath at 65 °C for one hour. Finally, it was washed with distilled water until the pH reached 7. The precipitate was collected and transferred to a vacuum drying oven at 65 °C until reached a constant weight. This will yield magnetic chitosan.

#### Preparation of magnetic chitosan/sludge biochar composite material

0.5 g of chitosan was weighed and dissolved in 250 mL of aqueous acetic acid solution with a volume fraction of 1%. The solution was stirred at room temperature until completely dissolved. The pH was adjusted to 6 with a NaOH solution of 20% by mass. Slowly poured the chitosan solution into 0.5 g of sludge biochar and placed it in a water bath at 60 °C with stirring for 4 h. After 4 h, slowly added 0.5 g of Fe_3_O_4_ magnetic fluid (prepared by dispersing 0.5 g of Fe_3_O_4_ particles in 100 mL of distilled water) to the mixture. The reaction was continued for 1 h. The resulting product was washed with distilled water until the supernatant was neutral. The product was placed in a vacuum drying oven at 65 °C and dried thoroughly. The dried product was thoroughly ground and stored for future use.

### Experimental methods

#### Determination of Cu^2+^ ions

This method is an improved double-ring ketone oxime (BCO) spectrophotometric method based on the dual aldehyde carbonyl oxime spectrophotometric method of China’s “Standard Test Method for Drinking Water” (GB/T5750-2006)^11,12^. In this method, aliquots of 0.0 mL, 0.1 mL, 0.2 mL, 0.4 mL, 0.8 mL, 1.2 mL, and 2.0 mL of the copper standard solution were transferred into seven 10 mL colorimetric tubes using a pipette. The color reagent was added sequentially according to the dual acetylacetone oxime (BCO) spectrophotometric method and then distilled water was added at the calibration line. The mixture was thoroughly mixed and allowed to stand for 15 min. The absorbance was measured using a visible spectrophotometer at a wavelength of 600 nm and a 5 cm cuvette with distilled water as reference. The Cu^2+^ standard curve was obtained by taking the measured absorbance as the vertical coordinate and the concentration of Cu^2+^ standard solution as the horizontal coordinate, from which the concentration of Cu^2+^ in the solution could be calculated.

#### Characterization of SBC and Fe_3_O_4_@CTS/SBC

Powdered sludge biochar (SBC) and magnetic chitosan-modified sludge biochar (Fe_3_O_4_@CTS/SBC) composites were characterized by X-ray diffractometer. The scanning angles for both samples were in the range of 10°–80° with a step size of 0.02° and a scanning speed of 10°/min. Fourier transform infrared (FTIR) spectroscopy was carried out on the samples of SBC, Fe_3_O_4_@CTS, Fe_3_O_4_@CTS/SBC in the range of wave numbers from500 cm^−1^ to 4000 cm^−1^. The morphology and structure of the samples were observed using a scanning electron microscope after dispersing the samples in ethanol solution at an accelerating voltage of 3 kV. The magnetic properties of the composite adsorbent were investigated by measuring the hysteresis return line of Fe_3_O_4_ @CTS/SBC using a vibrating sample magnetometer (VSM). The specific surface area and pore size analysis of the samples were performed under normal temperature and pressure conditions. The samples weighed 0.3 g and were subjected to N_2_ gas adsorption–desorption at a desorption temperature of 200 °C for approximately 6 h. The p/p_0_ values were in the range of 0.05 and 0.99, and BET calculations were used to determine the specific surface area. The pore size distribution of mesoporous materials was analyzed using the BJH model.

#### Single factor influence experiment

In this study, the effects of composite adsorbent dosage, initial concentration of Cu^2+^, pH and adsorption temperature on the adsorption effect were investigated. The fixed wastewater volume was 30 mL, the temperature of the shaking table was 25 °C, the rotational speed was 180r/min, and the adsorption time was 180 min, and the adsorption time was static. The supernatant was filtered with a 0.45um filter tip, and 2 mL of the filtrate was placed in a 10 mL colorimetric tube, and equal amounts of tri-ammonium citrate, pH 9 buffer, and BCO solution were added with the production standard. The absorbance was measured at 600 nm by visible spectrophotometer to determine the optimum conditions for adsorption of Cu^2+^.

### Analysis and calculation

The adsorption performance of the composites was evaluated using modified chitosan/sludge biochar as adsorbent. The effects of initial concentration of Cr (VI) ions, pH, temperature and adsorbent dosage on the adsorption performance of heavy metal ions were determined by batch tests. The adsorption rate (R) and adsorption capacity (q) were calculated using Eqs. (1) and (2).

1 $$R=\frac{{C}_{0}-{C}_{e}}{{C}_{0}}$$

2 $$Q=\frac{{(C}_{0-}{C}_{e})\mathrm{V}}{m}$$

where R is the adsorption rate, expressed as a percentage (%); Q is the adsorption capacity in mg/g; C_0_ represents the initial mass concentration of Cu^2+^ ions in the simulated wastewater in mg/L; C_e_ is the mass concentration of the equilibrium state of the Cu^2+^ ions after adsorption in mg/L; V represents the volume of simulated wastewater in mL; and m represents the mass of the adsorbent in grams (g).

#### Adsorption kinetics research

Adsorption kinetics models are used to investigate the mechanism of adsorption processes at the solid–liquid interface^13^. Commonly used kinetic models include pseudo-first-order adsorption rate equation^14^, pseudo-second-order adsorption rate equation^15^, and Weber-Morris intra-particle diffusion model^16^, which are used to explore the interaction mechanism between the adsorbent and the adsorbate.

Pseudo-first-order kinetic model equation:

3 $$\mathrm{ln}\left({q}_{e}-{q}_{t}\right)=ln{q}_{e,1}-{k}_{1}t$$

where q_e_ and q_t_ represent the adsorption amounts at equilibrium and at time t, respectively, in mg/g; q_e,1_ is the theoretical equilibrium adsorbed amount calculated from the pseudo-first-order kinetic rate equation in mg/g; k_1_ is the rate constant of the pseudo-first-order adsorption in min^−1^; plotting ln (q_e_ − q_t_) against t, the slope and intercept can be used to calculate k_1_ and q_e,1_.

Pseudo-second-order kinetic model equation:

4 $$\frac{t}{{q}_{t}}=\frac{1}{{k}_{2}{q}_{e,2}^{2}}+t/{q}_{e}$$

where q_t_ represents the adsorption amount at time t in mg/g; q_e,2_ represents the theoretical equilibrium adsorption amount calculated from the pseudo-second-order kinetic rate equation in mg/g; k_2_ is the rate constant of the pseudo-second-order adsorption in g/(mg·min). Plotting t on the x-axis and t/q_t_ on the y-axis, the slope and intercept can be used to calculate q_e,2_ and k_2_.

Weber-Morris intra-particle diffusion model:

5 $$ {\text{q}}_{{\text{t}}} = {\text{k}}_{{\text{p}}} {\text{t}}^{1/2} + {\text{C}} $$

where q_t_ represents the adsorption amount at time t in mg/g; k_p_ represents the intra-particle diffusion constant in mg/(g·min^1/2)^; C represents a constant related to the boundary layer thickness. Plotting t^1/2^ on the x-axis and qt on the y-axis, the slope and intercept can be used to calculate k_p_ and C. If the fitted line passes through the origin, it indicates that the adsorption rate is solely affected by intra-particle diffusion. If the line does not pass through the origin, it suggests that intra-particle diffusion is not the only rate-controlling step in the adsorption process.

Boyd first-order dynamics model. For intraparticle diffusion, Boyd^17^ derived the intraparticle diffusion model (Boyd pseudo first model). Boyd's intraparticle diffusion model is based on Fick’s second law of diffusion, which describes the change in particle concentration during diffusion. The model involves factors such as diffusion coefficients, diffusion rates and boundary conditions. According to different assumptions and boundary conditions, different forms of intraparticle diffusion models can be obtained. Boyd’s intraparticle diffusion model provides a quantitative analytical tool for studying the diffusion behavior of particles. It can help us understand and predict the concentration distribution, rate, and interaction with the environment and boundary conditions of particles during internal diffusion. The intraparticle diffusion model can be derived by solving the diffusion partial differential equation and making assumptions according to different situations, and its linearization formula is as follows:

6 $$\mathrm{ln}\left(1-{(\frac{q}{qe})}^{2}\right)={-k}_{i}t$$

where k_i_ represents the apparent adsorption rate parameter.

#### Adsorption isotherm model

The adsorption equilibrium relationship between the adsorbent and the adsorbate was described using the Langmuir isotherm model and the Freundlich model^18^. The Langmuir isotherm model is suitable for the monolayer adsorption with uniformly distributed energy adsorption sites, and the adsorption model is as follows^19^:

7 $$\frac{\mathrm{Ce}}{\mathrm{qe}}=\frac{1}{{\mathrm{q}}_{\mathrm{m}}{\mathrm{K}}_{\mathrm{L}}}+\mathrm{Ce}/{\mathrm{q}}_{\mathrm{m}}$$

where q_e_ represents the equilibrium adsorption capacity in mg/g; q_m_ represents the maximum adsorption capacity in mg/g; c_e_ represents the equilibrium concentration in mg/L; k_L_ represents the adsorption rate constant in L/mg.

The Freundlich isotherm model is suitable for the multilayer adsorption on adsorbents with heterogeneous physicochemical properties. The expression for the Freundlich isotherm model is as follows^20^:

8 $$\mathrm{lnqe}=\frac{1}{\mathrm{n}}\mathrm{lnCe}+{\mathrm{lnK}}_{\mathrm{F}}$$

where q_e_ represents the equilibrium adsorption capacity in mg/g; n is a constant related to the adsorption intensity; c_e_ represents the equilibrium concentration in mg/L; k_F_ represents the Freundlich adsorption rate constant in mg/g·(L/mg) ^(−1/n)^.

The Temkin isotherm is used to describe the chemisorption process based on electrostatic force of positive and negative charges, and its expression is as follows:

9 $${q}_{e}=\frac{\mathrm{RT}}{\mathrm{b}}ln{k}_{t}+\frac{\mathrm{RT}}{b}ln{c}_{e}$$

where q_e_ represents the equilibrium adsorption capacity in mg/g; c_e_ represents the equilibrium concentration in mg/L; kt is Temkin’s constant; b is the heat-related adsorption constant.

#### Adsorption thermodynamics study

Adsorption thermodynamics focuses on the conversion relationship between macroscopic thermal phenomena and other forms of energy^21^. The spontaneity of the adsorption process can be determined by the Gibbs free energy change (ΔG). When ΔG < 0, the adsorption process occurs spontaneously. When ΔG > 0, it is a non-spontaneous. Whether the adsorption process is exothermic or endothermic can be determined by the enthalpy change (ΔH). When ΔH < 0, the adsorption is exothermic in nature. On the contrary, it is heat-absorbing in nature. The Gibbs free energy change (ΔG), enthalpy change (ΔH) and entropy change (ΔS) are calculated by Eqs. (10) and (11).

10 $$\mathrm{lnk}=-\frac{\mathrm{\Delta H}}{\mathrm{RT}}+\frac{\mathrm{\Delta S}}{R}$$

11 $$\mathrm{\Delta G}=\mathrm{\Delta H}-\mathrm{T\Delta S}$$

where R represents the gas constant, 8.314 J/mol/k; T is the Kelvin temperature, K.

## Results

### Characterization of Adsorbent Materials

#### XRD analysis

**Figure 1:**
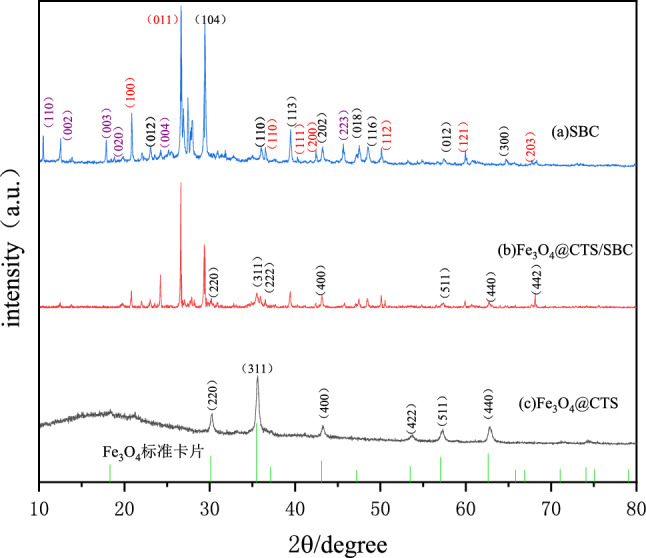
X-ray diffraction pattern of adsorbent.

XRD, also known as X-ray diffraction, is a technique used to analyze the crystal structure of different chemical substances. A unique x-ray diffraction pattern is produced by x-ray diffraction of the sample. In this study, SBC, Fe_3_O_4_@CTS and Fe_3_O_4_@CTS/SBC were analyzed by x-ray diffraction. The results are shown in Fig. 1.

Based on the XRD analysis, it could be determined that SBC mainly consists of calcite (CaCO_3_), quartz (SiO_2_), and cordierite (Mg, Fe_2_(Al_4_Si_5_O_18_)). The corresponding crystallographic indices (diffraction indices) for calcite are (012), (104), (110), (113), (202), (018), (116), (122), and (300); for quartz they are (100), (011), (110), (111), (200), (112), (121), and (203); and for cordierite they are (110), (002), (003), (020), (004), and (223). Therefore, these diffraction peaks could be observed in the XRD spectra of SBC and Fe_3_O_4_@CTS/SBC.

#### FT-IR analysis

**Figure 2:**
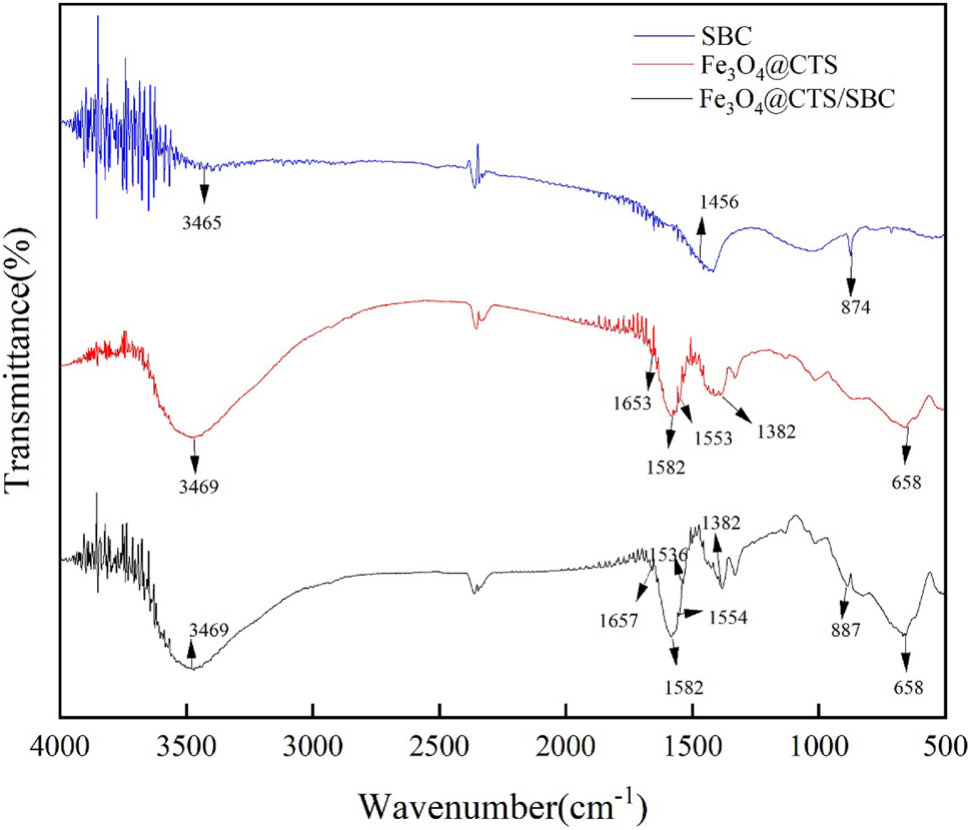
Infrared spectrogram of adsorbent.

FT-IR spectroscopy is used to study the structure of a sample and to determine the type of functional groups present. When a sample is exposed to infrared radiation of different wavelengths, it absorbs specific wavelengths, resulting in an infrared absorption spectrum of the substance^22^. The FT-IR spectra of SBC, Fe_3_O_4_@CTS, and Fe_3_O_4_@CTS/SBC composite adsorbents are shown in Fig. 2.

Sample SBC showed peaks at 874 cm^−1^ and 1456 cm^−1^, corresponding to the aromatic C-H and -COOH groups, respectively, which demonstrated that sludge biochar contains abundant oxygenated functional groups^23^. Moreover, sample Fe_3_O_4_@CTS/SBC also showed a peak at 887 cm^−1^, which indicated that the sludge biochar was formed by successful complexation with magnetic chitosan. Samples Fe_3_O_4_@CTS and Fe_3_O_4_@CTS/SBC showed peaks at 1382 cm^−1^, which could be attributed to the C–H deformation vibrations of chitosan's –CH_3_ group. The bending vibration absorption peak of -NH_2_ usually occurs in the range of 1650–1560 cm^−1^, and this functional group appeared at 1582 cm^−1^ in the infrared spectra of Fe_3_O_4_@CTS and Fe_3_O_4_@CTS/SBC, which indicated the presence of -NH_2_ bending vibration absorption peak in magnetic chitosan^24^, which confirmed the existence of chitosan in the composites. Sample Fe_3_O_4_@CTS exhibited a characteristic absorption peak of Schiff base (–C=N–) at 1653 cm^−1^, which indicated the participation of glutaraldehyde in cross-linking reaction. Both sample Fe_3_O_4_@CTS (1653 cm^−1^) and sample Fe_3_O_4_@CTS/SBC (1657 cm^−1^) showed characteristic absorption peaks corresponding to the stretching vibrations of the amide C=O group in Fe_3_O_4_@CTS, which indicated the presence of chitosan in the composites^25^. Peaks associated with the stretching vibration absorption of Fe–O appeared at 658 cm^−1^ in the spectra of Fe_3_O_4_@CTS and Fe_3_O_4_@CTS/SBC^26^, which indicated the successful formation of Fe_3_O_4_ on chitosan and the magnetic properties of Fe_3_O_4_@CTS/SBC.

#### SEM analysis

**Figure 3:**
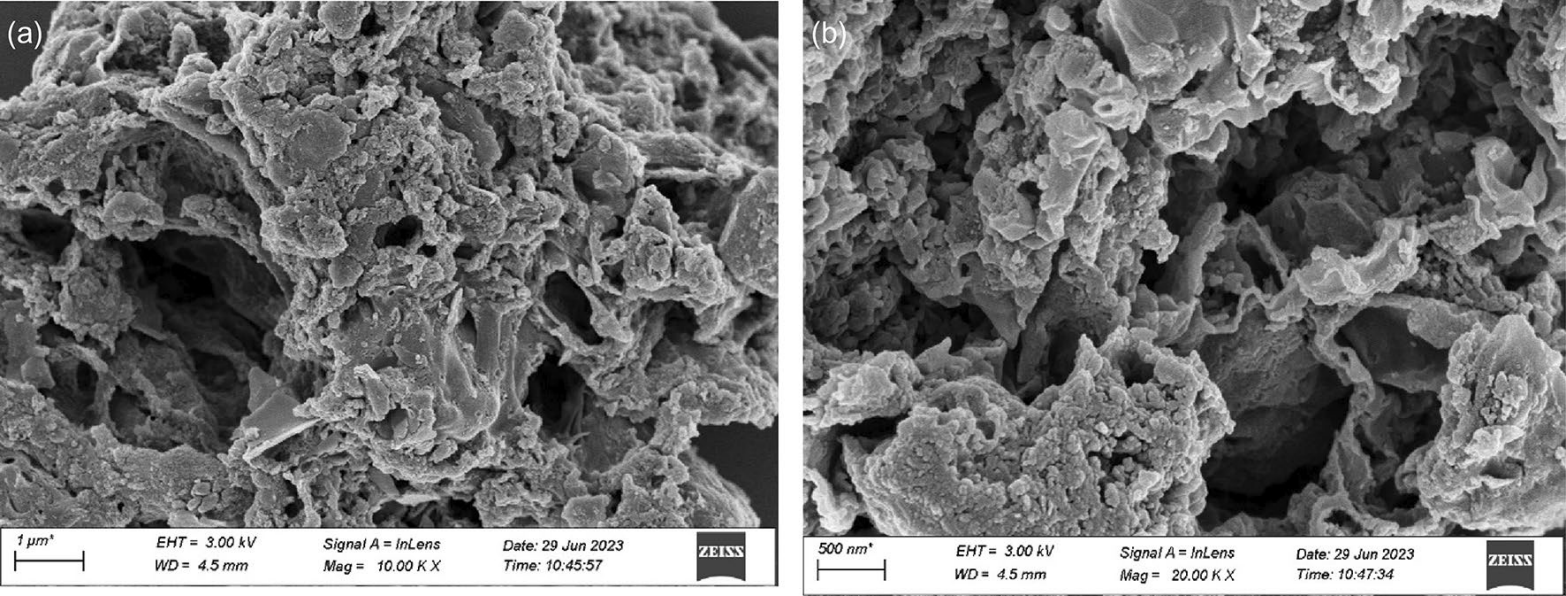
(**a**) SEM with 10,000 times SBC amplification, (**b**) SEM with 20,000 times SBC amplification.

**Figure 4:**
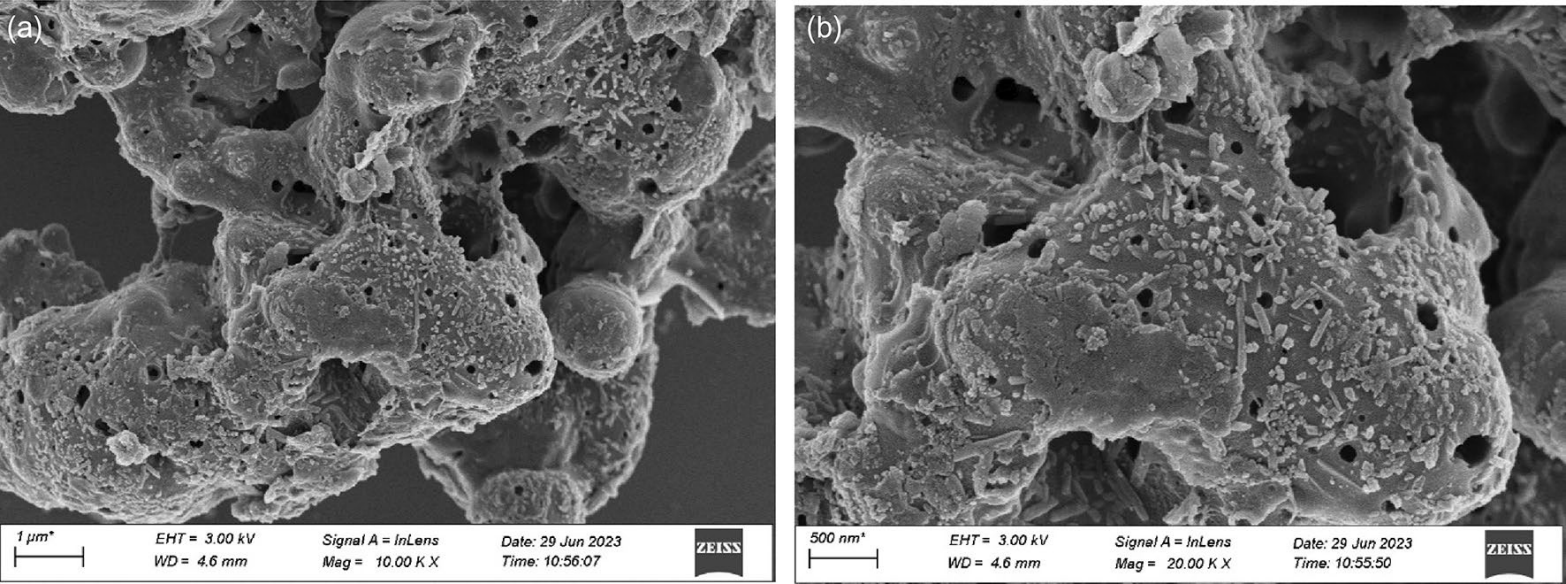
(**a**) Fe_3_O_4_@CTS/SBC SEM with 10,000 times magnification, (**b**) Fe_3_O_4_@CTS/SBC SEM with 20,000 times magnification.

The Scanning Electron Microscope (SEM) is commonly used for observing the surface morphology of materials. In order to gain a more intuitive and clear understanding of the microstructure of SBC and Fe_3_O_4_@CTS/SBC composite adsorbents, SEM analysis was performed on each sample. Figure 3a,b respectively showed the SEM images of SBC magnified 10,000 times and 20,000 times, while Fig. 4a,b respectively showed the SEM images of Fe_3_O_4_@CTS/SBC composite adsorbent magnified 10,000 times and 20,000 times.

#### Specific surface area and pore size analysis

**Figure 5:**
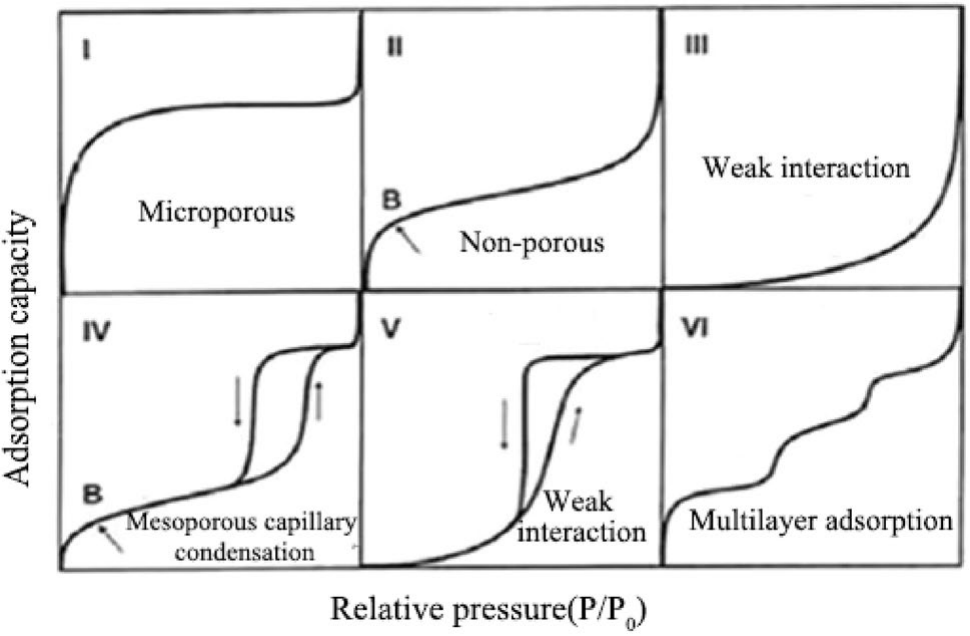
Adsorption isotherm type.

There are six common types of adsorption isotherms, as shown in Fig. 5. Type I is the most representative Langmuir isotherm, which is often observed in microporous materials. Type II is an S-shaped isotherm, mainly occurring on non-porous or macroporous solid surfaces. Type III is convex downward throughout the entire pressure range, with no inflection point. Type IV is similar to Type II, but in the low P/P_0_ region, the curve is convex upward. Unlike Type III, Type V is characterized by a convex shape towards the relative pressure axis, and the presence of an inflection point at higher relative pressures. Type VI is well known for its stepped adsorption process, where the stepped shape is attributed to the sequential multilayer adsorption on non-porous surfaces.

**Figure 6:**
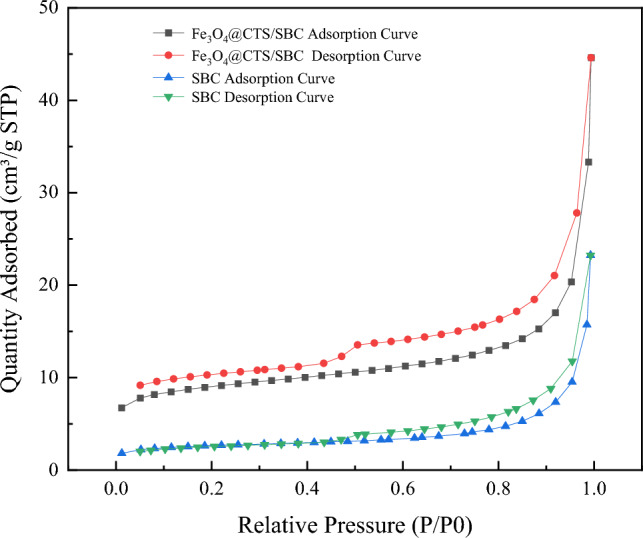
Adsorption and desorption isotherms of SBC and Fe_3_O_4_@CTS/SBC.

The N_2_ adsorption–desorption isotherms provide further insight into the properties of the adsorbent materials. In this study, the N_2_ adsorption–desorption isotherms of SBC and Fe_3_O_4_@ CTS/SBC composite adsorbents were shown in Fig. 6. Table 1 showed the specific surface area and pore volume of Fe_3_O_4_@CTS/SBC and SBC.

**Table 1:** Surface structure parameters of adsorbent.

Sample	Specific surface area/(m^2^/g)	Pore volume /(cm^3^/g)
	ABET	V_total_
SBC	9.33	0.0143
Fe_3_O_4_@CTS/SBC	32.86	0.0311

**Figure 7:**
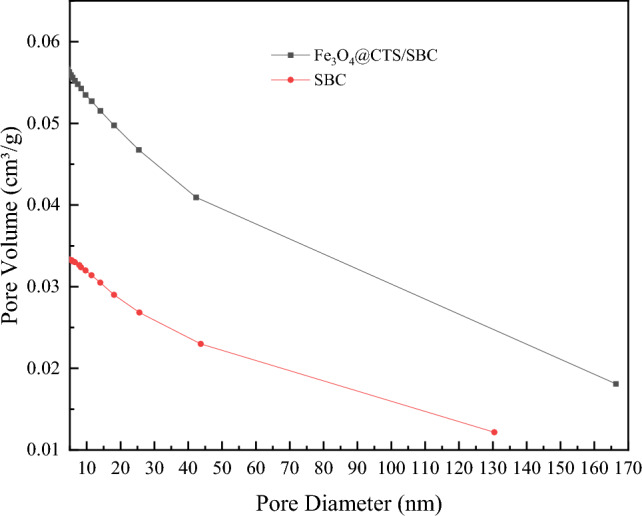
Aperture distribution of SBC and Fe_3_O_4_@CTS/SBC.

The pore size distribution of SBC and Fe_3_O_4_@CTS/SBC composite adsorbents could be observed from Fig. 7, which mainly lied between 2 to 50 nm, indicating that they are mesoporous materials. The figure also showed that the pore volume of the composite adsorbent is larger than that of sludge biochar.

#### Magnetic performance analysis

**Figure 8:**
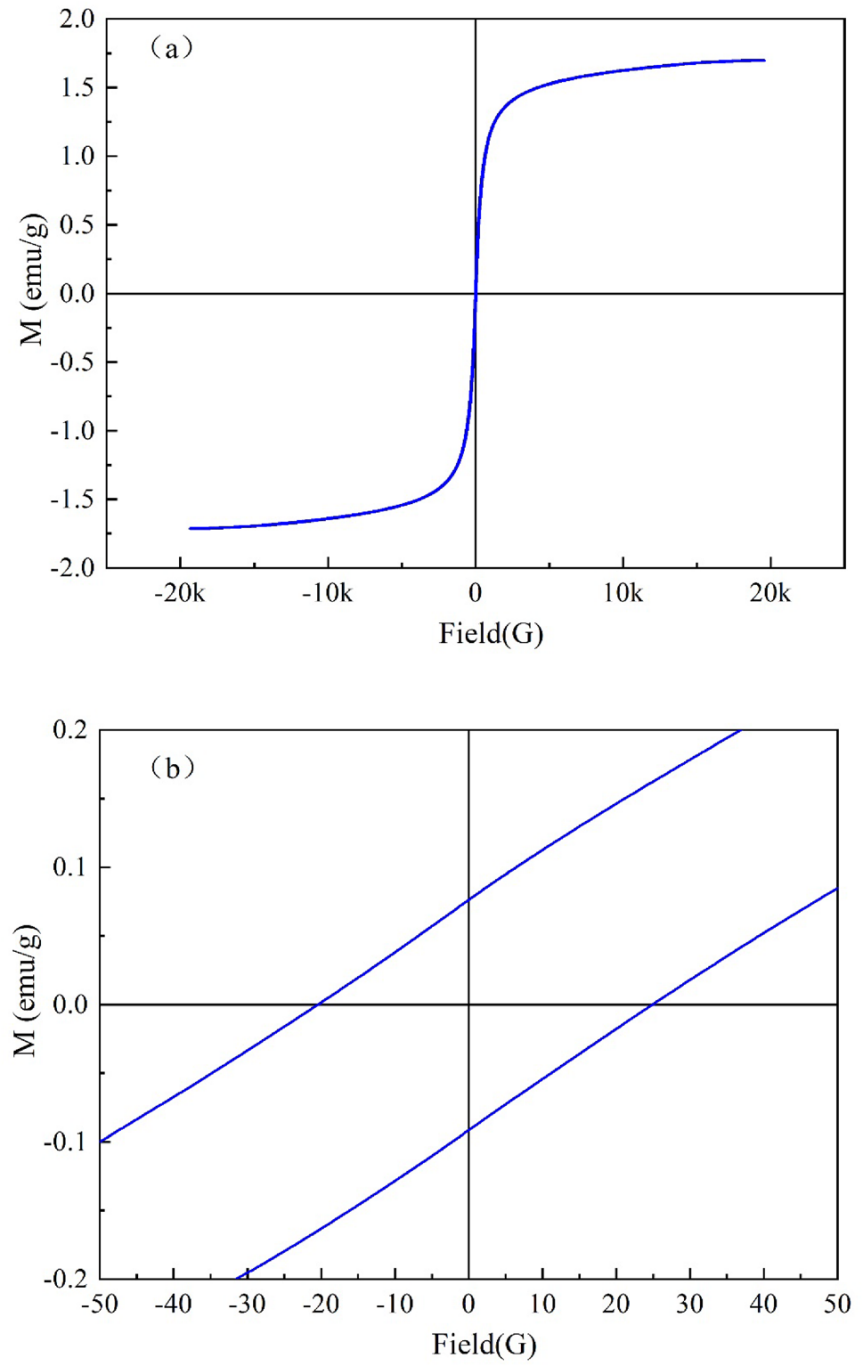
(**a**) Hysteresis loop of Fe_3_O_4_@CTS/SBC composite (**b**) coercivity curve of Fe_3_O_4_@CTS/SBC composite.

The magnetization intensity is an important factor for the solid–liquid separation after the adsorbent has completed the adsorption process. To study the magnetic properties of Fe_3_O_4_@CTS/SBC composites, the magnetic hysteresis line of Fe_3_O_4_@CTS/SBC was measured at room temperature, and the result were shown in Fig. 8a: The magnetic hysteresis line of Fe_3_O_4_@CTS/SBC composites showed an "S"-shaped curve. During the magnetization process, the magnetization intensity of the composites increased with the increased of the applied magnetic field strength and ultimately reached saturation. Fe_3_O_4_@CTS/SBC composites exhibited magnetism with a saturation magnetization intensity of 1.71 emu/g. Figure 8b showed that the composites had large coercivity of 25 Oe, which indicated strong ferromagnetism. The composites could be separated from the aqueous solution by applying an external magnetic field.

### Single factor impact experiment

**Figure 9:**
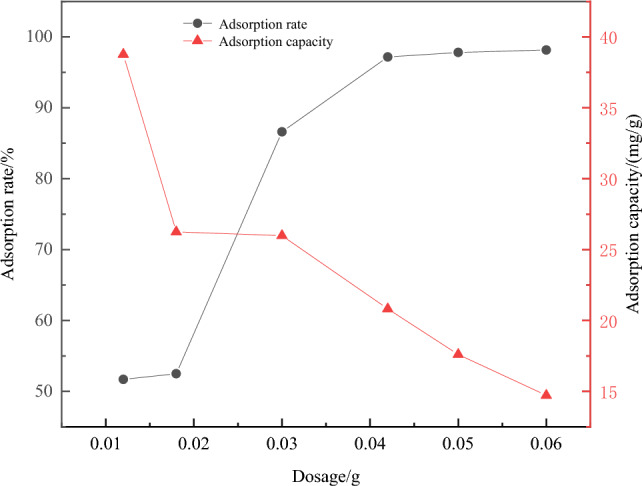
Influence of dosage on adsorption effect.

(1) The effect of Fe_3_O_4_@CTS/SBC composites dosage on Cu^2+^ adsorption efficiency: The experimental results were shown in Fig. 9.

According to Fig. 9, the adsorption efficiency of Fe_3_O_4_@CTS/SBC composites increased with the increased in the dosage of Fe_3_O_4_@CTS/SBC composites. However, when the dosage exceeded 0.03 g, the increased in adsorption efficiency became slower.

**Figure 10:**
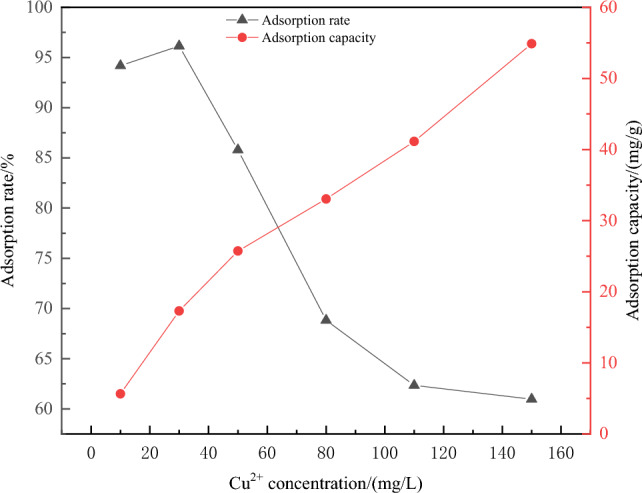
Effect of initial concentration of Cu^2+^ on adsorption effect.

(2) The effect of initial Cu^2+^ concentration on the adsorption performance: The experimental results were shown in Fig. 10.

From Fig. 10, the adsorption capacity of Fe_3_O_4_@CTS/SBC composites for Cu^2+^ increased with the increased of Cu^2+^ initial concentration. When the initial concentration of Cu^2+^ was lower than 30 mg/L, the adsorption efficiency showed an increasing trend and remained above 90%. The maximum value was reached when the initial concentration of Cu^2+^ was 30 mg/L.

**Figure 11:**
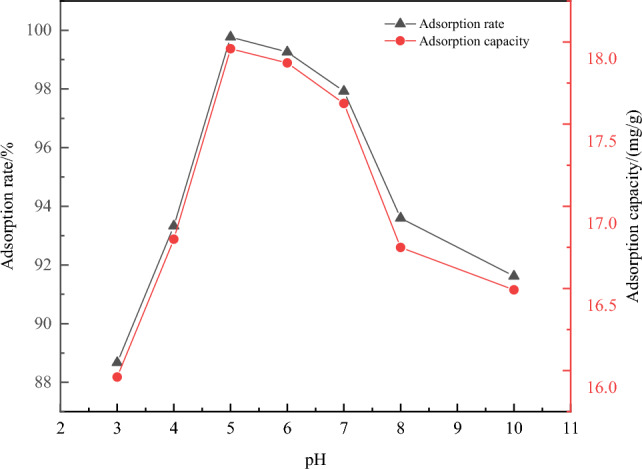
Effect of solution pH on adsorption of Cu^2+^.

(3) The effect of solution pH on adsorption efficiency of Cu^2+^ ions: The experimental results were shown in Fig. 11.

The pH of the solution can affect the surface charge of the adsorbent and the existence state of Cu^2+^ ions, thereby influencing the adsorption of Cu^2+^^27^. From the figure, it could be observed that the adsorption rate of Fe_3_O_4_@CTS/SBC composites Cu^2+^ in solution increased with the increased pH in the solution. The adsorption rate and adsorption capacity reached the maximum value at pH 5 and then began to decline.

**Figure 12:**
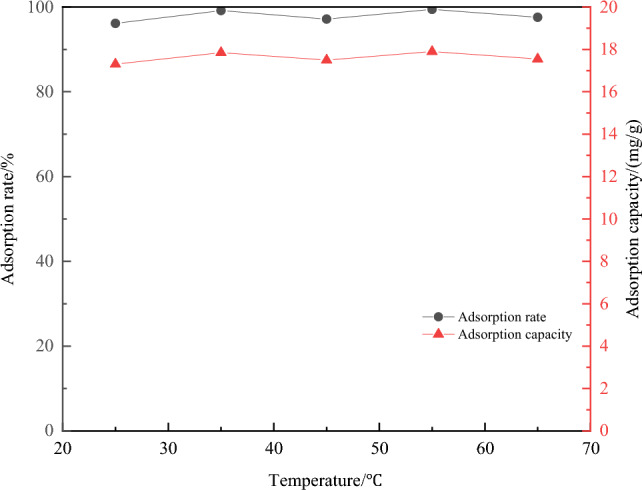
Effect of temperature on adsorption of Cu^2+^.

(4) The effect of temperature on the adsorption efficiency of Cu^2+^ ions: The experimental results were shown in Fig. 12.

From the figure, the adsorption efficiency of magnetic chitosan/sludge biochar was stable at about 90% with increasing temperature. The adsorption capacity also remained relatively stable. This indicated that the variation in temperature has little effect on the adsorption efficiency of the composites.

### Adsorption performance evaluation

#### Adsorption kinetics research

**Figure 13:**
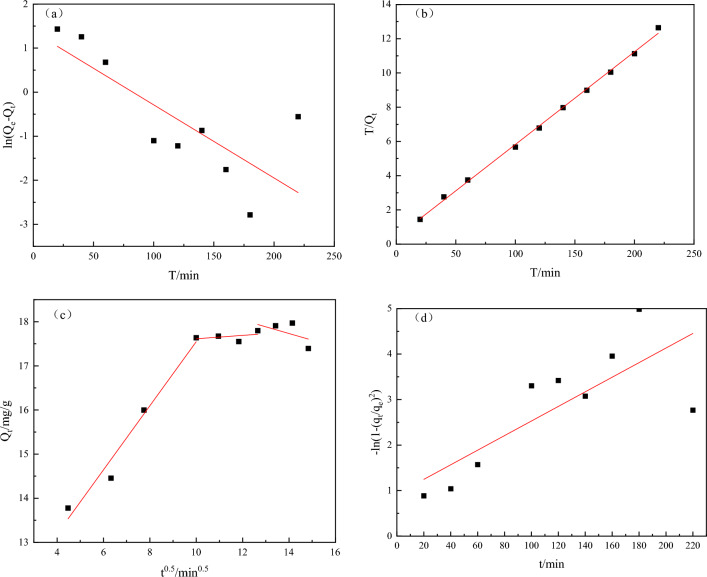
(**a**) Pseudo-first-order adsorption kinetic model (**b**) pseudo-second-order adsorption kinetic model, (**c**) Intraparticle diffusion model of Cu^2+^ adsorption (**d**) Boyd model of Cu^2+^ adsorption.

When the adsorption conditions were T = 298 K, pH = 5, Cu^2+^ concentration of 30 mg/L in 30 mL solution, and 0.5 g of Fe_3_O_4_@CTS/SBC composites, the experimental data were fitted by using pseudo-first-order, pseudo-second-order kinetic models, intraparticle diffusion model and Boyd first-order dynamics model. The specific results were shown in Fig. 13a–d.

For a copper sulfate solution with an initial concentration of 30 mg/L, the adsorption rate of Cu^2+^ was very fast within 100 min and reached adsorption equilibrium around 200 min. Each gram of Fe_3_O_4_@CTS/SBC composites could adsorb 17.9713 mg of Cu^2+^, at which point the equilibrium concentration of Cu^2+^ in solution was 0.17 mg/L. The adsorption kinetics of Cu^2+^ were shown in Fig. 13, and the fitting results were shown in Table 2. From Fig. 13 and Table 2, it could be observed that Qt/T showed a good linear relationship with T, and the R^2^ value of 0.9982. The saturated adsorption capacity was like to the experimental results, and the pseudo-second-order kinetics better was more in line with the adsorption of Cu^2+^ by the Fe_3_O_4_@CTS/SBC composite adsorbent.

**Table 2:** Comparison of kinetic models for adsorption of Cu^2+^ by Fe_3_O_4_@CTS/SBC complex adsorbent.

Adsorption kinetics models	
Simulating the concentration of Cu^2+^ (mg/L) in wastewater	30
Q_e,exp_/(mg/g)	17.9713
Pseudo-first-order kinetics	
k_1_/(min^−1^)	0.0166
Q_e_/(mg/g)	3.9476
R^2^	0.6169
Pseudo-second-order kinetics	
k_2_/(g/(mg min))	0.0072
Q_e_/(mg/g)	18.4672
R^2^	0.9982
Intraparticle diffusion model	
k_p,1_	0.7264
k_p,2_	0.0387
k_p,3_	− 0.1518
Boyd first-order dynamics model	
k_i_	0.01603
R^2^	0.6106

The intraparticle diffusion model was fitted with t^0.5^ as the x-coordinate and Q_t_ as the y-coordinate. The fitting results were shown in Fig. 13c. The line in the figure did not pass through the origin, indicating that intra-particle diffusion was not the only step controlling the adsorption rate. Therefore, the adsorption of Cu^2+^ on Fe_3_O_4_@CTS/SBC composite adsorbent could be divided into three stages. According to the fitting data in Table 2, the adsorption rate slowed down with increasing adsorption time.

The adsorption capacity of copper ions by 0.5 g composites combined with the linearized adsorption kinetics model (Eq. (6)), linearized fitted adsorption kinetic model could be obtained respectively (Fig. 13d, and the relevant parameters of the fitted line were shown in Table 2.

#### Adsorption isotherm research

**Figure 14:**
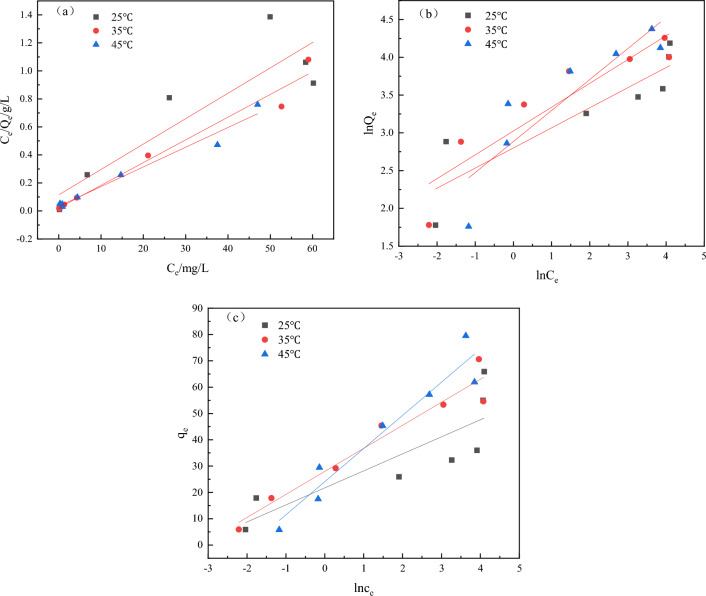
Fe_3_O_4_@CTS/SBC (**a**) Langmuir (**b**) Freundlich (**c**) Temkin isotherm model for Cu^2+^.

The adsorption isotherms of Fe_3_O_4_@CTS/SBC composite adsorbent were investigated for Cu^2+^ adsorption at constant temperature. The experiments were carried out at 25 °C, 35 °C, and 45 °C, respectively. The Langmuir model, Freundlich model and Temkin model were used to fit the adsorption process. The initial concentration of Cu^2+^ was chosen to be in the range of 10–170 mg/L. Other adsorption conditions included pH 5, 0.5 g Fe_3_O_4_@CTS/SBC composite adsorbent. The adsorption isotherms of Cu^2+^ were shown in Fig. 14a–c, and the fitted results were presented in Table 3.

**Table 3:** Langmuir, Freundlich and Temkin isotherm parameters of Cu^2+^ adsorption.

The isotherm equation	Parameters	Temperature/(K)		
		298	308	318
Langmuir	Q_m_/(mg/g)	55.16	61.65	71.07
	K_L_/(L/mg)	0.1579	0.7874	0.4268
	R^2^	0.8288	0.9735	0.9719
Freundlich	K_F_/(mg^1–1/n^L^1/n^/g)	16.441	20.579	17.895
	n	3.76	3.17	2.44
	R^2^	0.8081	0.8531	0.8108
Temkin	Kt	28.82	24.51	6.787
	b	375	286	205
	R^2^	0.7199	0.9421	0.9272

The results showed that although the equilibrium concentration of Cu^2+^ (C_e_) increased with the initial concentration, the equilibrium adsorption capacity (Q_e_) of the adsorbent also increased. Linear regression analyses were performed on the isotherm data, and Fig. 14a,b showed the linear relationships between C_e_/Q_e_ and C_e_, as well as lnQ_e_ and lnC_e_. In Fig. 14a, Q_m_ and K_L_ could be calculated, and the correlation coefficients R^2^ at different temperatures were 0.8288, 0.9735, and 0.9719, respectively, which were all closer to 1 than the Freundlich model. In this study, the Langmuir model was more suitable for the adsorption of Cu^2+^ by the Fe_3_O_4_@CTS/SBC composite adsorbent. The average saturation adsorption capacities at the three temperatures were 65.89 mg/g, 70.60 mg/g, and 79.49 mg/g, which were very close to the calculated maximum adsorption capacities of 55.16 mg/g, 61.65 mg/g, and 71.07 mg/g, respectively. In addition, the Freundlich isotherm was fitted with n greater than 1, indicating that the adsorption process was easy to carry out. The Temkin isotherm model was well fitted, which indicated that the presence of electrostatic force for the adsorption of Cu^2+^ by Fe_3_O_4_@CTS/SBC.

#### Adsorption thermodynamics research

The slope and intercept of the straight line obtained by lnk plotting 1/T gives ΔH and ΔS, and then ΔG. The results were shown in Table 4.

**Table 4:** Thermodynamic parameter.

T/K	Thermodynamic parameter		
	ΔG/(kJ/mol)	ΔH/(kJ/mol)	ΔS/(kJ/mol/K)
298	− 25.55	3.949	0.099
308	− 26.54		
318	− 27.53		

As shown in Table 4, ΔG < 0, ΔH > 0 of Fe_3_O_4_@CTS/SBC adsorbent for Cu^2+^ indicated that the adsorption process was a spontaneous endothermic reaction. ΔS was greater than 0, which indicated that the adsorption of Cu^2+^ by the adsorbent was a process of entropy increased, and the chaos of the solid–liquid interface increased.

## Discussion

### Instrumental analysis

In the XRD analysis of this study, the six diffraction peaks of Fe_3_O_4_@CTS corresponding to (2θ = 30.1°, 35.5°, 43.1°, 53.5°, 57.0°, and 62.6°) by comparing them with the PDF standard card of Fe_3_O_4_ represent (220), (311), (400), (422), (511), and (440) respectively of the crystal surface^28,29^. This indicated that magnetite has been successfully loaded onto the chitosan surface and magnetic chitosan has been successfully prepared. From the XRD spectrum of Fe_3_O_4_@CTS/SBC, it could be seen that the Fe_3_O_4_@CTS/SBC composites also exhibited the same diffraction peaks as Fe_3_O_4_@CTS material. Additionally, the characteristic peaks of Fe_3_O_4_ remained at 37.1° and 66.9°, which indicated that Fe_3_O_4_ has been loaded onto the SBC. Furthermore, weak peaks were observed at 25.9° and 28.2°, which correspond to the diffraction peaks of chitosan (CTS) on the standard card, which indicated that CTS has been loaded onto SBC. This demonstrated the successful preparation of the Fe_3_O_4_@CTS/SBC composites. The XRD spectrum of the Fe_3_O_4_@CTS /SBC composites retained the main diffraction peaks of SBC, but the intensity was changed. This was due to the loading of CTS and Fe_3_O_4_ onto the surface of sludge biochar.

From the SEM analysis of this study, from Fig. 3, it could be observed that sludge biochar (SBC) had a rough wrinkled surface appearance and an abundant pore structure. This is due to the pore structure formed during the pyrolysis process in the tubular furnace, which gave SBC a certain adsorption capacity. Figure 4 showed that the morphology of the composites changed significantly compared to SBC. The Fe_3_O_4_@CTS/SBC had a rough and porous surface with fewer macropores and a predominantly mesoporous structure. Visible white particles of magnetic nanoparticles were attached to the surface of SBC, which were adhered to the surface of SBC through chitosan. This indicated that Fe_3_O_4_ had been successfully loaded onto the surface of the biochar. These particles did not appear to be significantly aggregated, which indicated that the Fe_3_O_4_ particles could be uniformly dispersed on the biochar surface. The biochar could serve as an effective carrier, which was more conducive to adsorption.

The analysis of the adsorption–desorption isotherms using inert N_2_ gas on SBC and Fe_3_O_4_@CTS/SBC adsorbents indicated that both exhibited Type IV adsorption isotherms, which were typical for mesoporous materials. Additionally, a H_2_-type hysteresis return lines were observed. When P/P_0_ < 0.4, SBC showed monolayer adsorption of N_2_, and the inflection point was followed by the end of monolayer adsorption and the beginning of multilayer adsorption. It could also be observed from the figure that the adsorption capacity of SBC on N_2_ was low, while the adsorption capacity of Fe_3_O_4_@CTS/SBC composite adsorbent was high. This indicated that the specific surface area of the composite adsorbent had increased.

According to Table 1, the specific surface area of Fe_3_O_4_@CTS/SBC was 3.52 times larger than that of SBC, and the pore volume was increased by 2.17 times. The improvement in specific surface area was highly beneficial for enhancing the adsorption capacity of Fe_3_O_4_@CTS/SBC. Furthermore, the introduction of chitosan introduced active functional groups such as -NH_2_ and -OH on the surface of the composite adsorbent, which further enhanced the interaction between the adsorbent and heavy metal ions.

### Effect of parameters

From Fig. 9, with the increase in the dosage of the composites, the number of active groups bound to Cu^2+^ also increased, leading to an increase in the adsorption rate. However, when the dosage exceeded 0.03 g, the concentration of Cu^2+^ remained constant and the number of active groups involved in binding Cu^2+^ reached a critical value, resulting in a less significant increase in the adsorption efficiency^30^. At the same time, it could be observed from the figure that the adsorption capacity of Cu^2+^ gradually decreased with the increased in the dosage of the material. This was because when the dosage of the adsorbent was low, the adsorption capacity of Fe_3_O_4_@CTS/SBC composites for Cu^2+^ quickly reached saturation. However, continuing to increase the dosage leaded to more vacant binding sites on the surface of the adsorbent due to the fixed amount of adsorbate, which leaded to a decrease in the adsorption capacity. Based on the above analysis, 0.05 g was selected as the optimal dosage in this study.

As can be seen from Fig. 10, the adsorption capacity of Fe_3_O_4_@CTS/SBC composites for Cu^2+^ increased with the increased in the initial concentration of Cu^2+^. This was because as the initial concentration of Cu^2+^ increased, there were more adsorbates, resulting in a larger concentration difference between the solid and liquid phases, which provided a driving force for adsorption. In addition, with the increased of adsorbate quantity, the driving force increased, leading to an increase in the attraction between the adsorbate and adsorbent, which increased the adsorption capacity^31^. The adsorption efficiency showed an increasing trend when the Cu^2+^ initial concentration was lower than 30 mg/L, and it remained above 90%. The maximum point was reached at the initial concentration of Cu^2+^ of 30 mg/L. However, when the initial concentration of Cu^2+^ exceeded 30 mg/L, the adsorption efficiency of the material gradually decreased. This was because at lower Cu^2+^ concentrations, Fe_3_O_4_@CTS/SBC composites provided more adsorption sites, which resulted in better adsorption efficiency. However, with the increased of Cu^2+^ content in the solution, the adsorption sites on the surface of Fe_3_O_4_@CTS/SBC composites gradually decreased during the adsorption process at a certain adsorbent addition amount. When the adsorption sites of the composites reached saturation, it no longer adsorbed Cu^2+^, but the initial concentration of Cu^2+^ continued to increase. Therefore, the adsorption efficiency of the magnetic chitosan/sludge biochar composites decreased with the increased of the initial concentration of Cu^2+^. In summary, the initial concentration of Cu^2+^ of 30 mg/L should be selected for subsequent experiments.

From Fig. 11, under acidic conditions, the amino groups of chitosan in the Fe_3_O_4_@CTS/SBC composites mainly existed in the form of -NH_3_^+^, which repelled the positively charged divalent Cu^2+^ ions^32^. Meanwhile, under acidic conditions, the concentration of H^+^ in water significantly increased. H^+^ competed for the adsorption sites of the adsorbent with Cu^2+^, which weakened the complexing ability of the composites towards Cu^2+^ ions^33^. Therefore, under acidic conditions, the concentration of H^+^ in water gradually decreased with the increase of pH, which leaded to the increase of adsorption efficiency. When the pH was greater than 6, Cu^2+^ ions in the copper solution easily formed precipitates in the form of Cu (OH)_2_, which leaded to a decrease in the adsorption efficiency. When the pH approached 7, chitosan was prone to coagulation, making it difficult to adsorb copper ions and resulting in a decrease in adsorption efficiency^34^. Comparative experiments showed that when the pH of the solution was less than 6, Cu^2+^ was evenly distributed in the solution, and the magnetic composite material had a good adsorption performance for copper ions. When the pH was greater than 7, Cu (OH)_2_ precipitation would be formed, which interfered with the adsorption process and the adsorption performance began to decline. Therefore, based on the experimental data, the optimum pH for the adsorption of Cu^2+^ by the magnetic chitosan/sludge biochar composites was 5.

Comparative experiments showed that under the optimum conditions (initial concentration of Cu^2+^ = 30 mg/L, dosage of adsorbent = 0.05 g, solution pH 5, temperature = 25 °C), the removal of Cu^2+^ by Fe_3_O_4_@CTS/SBC was 99.77%. The maximum adsorption capacity was 55.16 mg/g, while the removal rates of Fe_3_O_4_@CTS and SBC for Cu^2+^ were 80.63% and 77.54%, respectively, which was much lower than the adsorption performance of the composites for copper ions, which indicated that the materials prepared in this experiment had better performance.

### Adsorption kinetic analysis

The adsorption kinetics analysis indicated that the steps controlling the adsorption rate may be limited by chemisorption, which involved the sharing or exchanging electrons between the adsorbent and the adsorbate^35^. The driving force of this process depended on the square of the adsorption sites^31^, which could explain the rapid adsorption rate at the initial stage of adsorption. However, in the later stage of adsorption, the reduction of adsorption sites on the Fe_3_O_4_@CTS/SBC composite adsorbent and the decreased in Cu^2+^ concentration leaded to a decrease in the chemical driving force, which resulted in a slower adsorption rate^36^.

The slopes of the three segments in the broken line were the particle diffusion rate constants k_p,1_ > k_p,2_ > k_p,3_. The adsorption process of Cu^2+^ on Fe_3_O_4_@CTS/SBC composite adsorbent could be described as follows: in the first stage of adsorption, Cu^2+^ combines with the adsorptive active sites of the functional groups on the surface of the Fe_3_O_4_@CTS/SBC composite adsorbent, and the initial adsorption rate was fast^37^. When the number of surface-active sites gradually decreased, it entered the second stage of adsorption. The adsorption sites on the surface of the Fe_3_O_4_@CTS/SBC composite adsorbent were mainly occupied by Cu^2+^, which began to enter the micropores of the composite adsorbent and was adsorbed by the micropores. At the third stage of adsorption, the adsorption sites on the surface and inside the pores of the Fe_3_O_4_@CTS/SBC composite adsorbent were basically saturated, and the adsorption reached an equilibrium state^38^. Based on the particle diffusion model, it could be concluded that the adsorption of Cu^2+^ by the Fe_3_O_4_@CTS/SBC composite adsorbent was not only a chemical chelation, but also an intraparticle diffusion process.

The Boyd model is based on intraparticle diffusion rate control^39^. The R^2^ of Table 2 was 0.6106, which indicated that the model was poorly correlated. The intraparticle diffusion was not the only rate control step in the adsorption process of the Fe_3_O_4_@CTS/SBC on copper ions, which was consistent with the findings of the intraparticle diffusion model. The Boyd model did not describe the adsorption of the Fe_3_O_4_@CTS/SBC on copper ions well. In conclusion, the intraparticle diffusion model and the Boyd model can be useful to identify reaction pathways and adsorption mechanisms as well as to predict the rate-controlling step.

### Adsorption isotherm analysis

The Freundlich model is more suitable for adsorption on highly heterogeneous surfaces lacking a saturated adsorption platform, in which case inhomogeneous processes occurred on the adsorbed surfaces and the adsorption process involved adsorption in multiple molecular layers^7^. The adsorption process in this study followed the Langmuir model. The Langmuir model assumes a monolayer adsorption effect, thus the Fe_3_O_4_@CTS/SBC composite adsorbent exhibited monolayer adsorption, where only one molecule could be adsorbed at each active site, and all the adsorption sites had the same priority to form a homogeneous monolayer adsorption film^40^.

Table 5 compared the removal effect of Fe_3_O_4_@CTS/SBC composite adsorbent with the other adsorbents for the removal of Cu^2+^ under the room temperature conditions. Obviously, the Fe_3_O_4_@CTS/SBC composite adsorbent showed better adsorption of 55.16 mg/g, which exceeded that of magnetite/carbon nanocomposites (41.11 mg/g)^41^, CS-SiO_2_@TEuTTA luminous film (51.28 mg/g)^42^, DETA/SiO_2_/Fe_3_O_4_ (13.459 mg/g)^43^ and Dredged sludge-based adsorbent (40.62 mg/g)^44^. Although there were adsorbents with excellent adsorption properties such as porous poly (lactic acid)/chitosan nanofibers (111.66 mg/g)^45^, PS/Fe_3_O_4_/CS-PEI (204.6 mg/g)^46^ and MCA-MCS-TiO_2_ (220.67 mg/g)^47^, these adsorbents were not favorable for production and application due to their complicated preparation process and high cost.

**Table 5:** Comparison of Cu^2+^ adsorption by Fe_3_O_4_@CTS/SBC complex adsorbent and other adsorbents.

Adsorbent	Adsorption capacity/(mg/g)	References
Porous polylactic acid/chitosan nanofiber	111.66	Zia et al.^45^
Magnetite/carbon nanocomposite material	41.11	Andelescu et al.^41^
Luminescent film CS-SiO_2_@TEuTTA	51.28	Li et al.^42^
DETA/SiO_2_/Fe_3_O_4_	13.459	Chen et al.^43^
Dredged sludge-based adsorbent	40.62	Zhang et al.^44^
PS/Fe_3_O_4_/CS-PEI	204.6	Xiao et al.^46^
MCA-MCS-TiO_2_	220.67	Yu et al.^47^
Fe_3_O_4_@CTS/SBC composite adsorbent	55.16	This study

### Adsorption thermodynamic analysis

Literature^48,49^ suggested that the adsorbent-adsorbent interaction forces were hydrogen bonding, dipole attraction, van der Waals forces and chemical bonding when the values of |ΔH are 2 ~ 40 kJ/mol, 2 ~ 29 kJ/mol, 4 ~ 10 kJ/mol, and greater than 60 kJ/mol, respectively. When the ΔG values were between − 20 ~ 0 kJ/mol and − 80 ~ 400 kJ/mol, the adsorption was physical adsorption and chemical adsorption, respectively. As can be seen from Table 4, the |ΔH| adsorbed by Fe_3_O_4_@CTS/SBC on Cu^2+^ is 3.949 kJ/mol, which indicated that there were hydrogen bonding and dipole moment attraction between Fe_3_O_4_@CTS/SBC adsorbent and Cu^2+^. The ΔG values for Cu^2+^ took values ranging from − 25.55 to − 27.53 kJ/mol, which indicated that the adsorption of Cu^2+^ by Fe_3_O_4_@CTS/SBC adsorbent was chemisorption.

## Conclusion

In this study, a novel magnetic and recyclable biochar adsorbent was successfully synthesized using chitosan, Fe_3_O_4_, and sludge biochar as raw materials. The magnetic chitosan/sludge biochar composite adsorbent exhibited excellent adsorption performance for Cu^2+^. The maximum removal rate of 99.77% was achieved at an initial Cu^2+^ concentration of 30 mg/L, a dosage of 0.05 g of magnetic chitosan/sludge biochar composite, an adsorption time of 180 min, and a pH value of 5. Additionally, the maximum adsorption capacity was determined to be 55.16 mg/g at room temperature. The adsorption kinetics of Cu^2+^ by the magnetic chitosan/sludge biochar composite adsorbent followed the pseudo-second-order kinetic model. The adsorption isotherm conformed to the Langmuir isotherm model, which indicated that the adsorption process was monolayer chemisorption. As a novel and highly efficient adsorbent, Fe_3_O_4_@CTS/SBC has a great potential for the treatment of Cu^2+^ polluted wastewater. It has a wide material sources and economical preparation cost, making it a desirable option. Future research will focus on testing the effectiveness of the Fe_3_O_4_@CTS/SBC on other toxic heavy metals and metalloids, as well as mixed contaminants, through pilot-scaled tests. Additionally, the recovery performance of the adsorbent will be studied through regeneration experiments to evaluate its sustainability and reusability.

### Supplementary Information

Supplementary Information.
